# S-Adenosyl-Methionine and Betaine Improve Early Virological Response in Chronic Hepatitis C Patients with Previous Nonresponse

**DOI:** 10.1371/journal.pone.0015492

**Published:** 2010-11-08

**Authors:** Magdalena Filipowicz, Christine Bernsmeier, Luigi Terracciano, Francois H. T. Duong, Markus H. Heim

**Affiliations:** 1 Hepatology Laboratory, Department of Biomedicine, University Hospital Basel, Basel, Switzerland; 2 Department of Gastroenterology and Hepatology, University Hospital Basel, Basel, Switzerland; 3 Institute for Pathology, University Hospital Basel, Basel, Switzerland; Saint Louis University, United States of America

## Abstract

**Background/Aims:**

Treatment of chronic hepatitis C (CHC) with pegylated interferon α (pegIFNα) and ribavirin results in a sustained response in approximately half of patients. Viral interference with IFNα signal transduction through the Jak-STAT pathway might be an important factor underlying treatment failure. S-adenosyl-L-methionine (SAMe) and betaine potentiate IFNα signaling in cultured cells that express hepatitis C virus (HCV) proteins, and enhance the inhibitory effect of IFNα on HCV replicons. We have performed a clinical study with the aim to evaluate efficacy and safety of the addition of SAMe and betaine to treatment of CHC with pegIFNα/ribavirin.

**Methods:**

In this open-label pilot study, 29 patients with CHC who failed previous therapy with (peg)IFNα/ribavirin were treated with SAMe, betaine, pegIFNα2b and ribavirin. Treatment duration was 6 or 12 months, depending on genotype, and the protocol comprised a stopping rule at week 12 if early virological response (EVR) was not achieved. Virological and biochemical response and safety were assessed throughout the treatment.

**Results:**

29 patients were enrolled and treated according to the study protocol. 79% of the patients were infected with genotype 1, 72% had advanced fibrosis, 76% had previously received pegIFNα/ribavirin, and only 14% achieved EVR to the previous treatment. When treated with the study medications, 17 patients (59%) showed an EVR, only 3 (10%) however achieved a sustained virological response (SVR). SAMe and betaine were found to be safe when used with pegIFNα/ribavirin.

**Conclusion:**

The addition of SAMe and betaine to pegIFNα/ribavirin improves early virological response in CHC.

**Trial Registration:**

ClinicalTrials.gov NCT00310336

## Introduction

Hepatitis C virus (HCV) infection is a major cause of chronic liver disease worldwide [1]. Chronic hepatitis C (CHC) may lead to liver cirrhosis and hepatocellular carcinoma. Type I interferons (IFNs), IFNα and IFNβ, are crucial and potent components of the early host response against virus infection [2] and recombinant (pegylated) IFNα2a and IFNα2b are widely used for the treatment of CHC and chronic hepatitis B. The current standard treatment of CHC with pegylated IFNα (pegIFNα) and ribavirin leads to cure in about 50% of patients [3], [4]. The cause of treatment failure in the remaining half of the patients is poorly understood. Viral interference with IFNα signal transduction from the cell surface to the nucleus is considered an important mechanism behind ineffective treatment responses.

IFNα binds to the IFNα receptor (IFNAR) on the cell surface and triggers a signaling pathway that involves the Janus kinases (Jaks) Jak1 and Tyk2 and the signal transducer and activator of transcription (STAT) 1, STAT2 and STAT3 [5]. IFNα signaling through the Jak-STAT pathway ultimately results in transcriptional upregulation of IFN-stimulated genes (ISGs) with potent antiviral, immunomodulatory and antiproliferative properties. We have previously shown that expression of HCV proteins in human osteosarcoma cell lines inhibits IFNα signaling by an upregulation of the catalytic subunit of protein phosphatase 2A (PP2Ac) [6], [7], [8]. In line with our *in vitro* findings, PP2Ac was also over-expressed in liver extracts of HCV transgenic mice and in liver biopsies of patients with CHC [8]. An important consequence of the increased amount of PP2Ac is the inhibition of protein arginine methyltransferase 1 (PRMT1) [8], [9]. PRMT1 catalyzes methylation of STAT1 [10] as well as of PIAS1 (protein inhibitor of activated STAT1) [11]. Therefore, inhibition of PRMT1 by PP2Ac has important consequences for the Jak-STAT signaling pathway.

S-adenosyl-L-methionine (SAMe) is the methyl group donor for reactions catalyzed by PRMT1. Betaine is required for the generation of methionine from homocysteine, a reaction that is central to the recycling of SAMe [12]. We have previously shown that treatment with SAMe and betaine potentiates IFNα signaling and enhances the anti-viral efficacy of IFNα in osteosarcoma cells expressing HCV proteins and in human hepatoma Huh7 cells harboring an HCV replicon [13]. Furthermore, NS3 helicase activity and replication of a subgenomic HCV replicon in Huh7.5 cells were found to be inhibited by SAMe treatment [9].

Based on these *in vitro* findings we hypothesized that addition of SAMe and betaine to the current standard therapy with pegIFNα and ribavirin enhances the treatment efficacy in CHC patients with an impaired IFNα signal transduction, notably in previous nonresponders to (peg)IFNα/ribavirin.

## Materials and Methods

### Patients

The protocol for this trial and supporting CONSORT checklist are available as supporting information; see Checklist S1 and Protocol S1. Male and female patients between 18 and 65 years with CHC of all HCV genotypes (GTs) and a documented nonresponse to previous combination treatment with unmodified IFNα or pegIFNα plus ribavirin were eligible for enrollment. Eligible patients were identified by a systematic review of patient charts at the hepatology outpatient clinic of the University Hospital of Basel, Switzerland. Eligible patients had detectable HCV RNA in the serum (>12 IU/ml), elevated serum alanine aminotransferase (ALAT) levels, a liver biopsy taken within 2 years before the screening visit showing chronic hepatitis and compensated liver disease. Entry neutrophil and platelet counts had to be at least 1500/µl and 75000/µl, and hemoglobin values at least 110 g/l. Patients with the following criteria were excluded: any other cause of liver disease, co-infection with human immunodeficiency or hepatitis B virus, pregnant women and male partners of pregnant women, breast feeding women, fertile patients not willing to use effective contraception, concomitant immunosuppressive medication, excessive alcohol intake, concomitant malignant neoplastic disease, clinically significant psychiatric, metabolic, neurological, cardiac, renal or endocrine disease, anti-nuclear antibody titer >1∶320, α-fetoprotein >50 µg/l, elevated fasting glucose level, elevated thyroid-stimulating hormone.

### Study Medication

SAMe (Gumbaral 200 mg) was purchased from AWD.pharma GmbH & Co, D-01445 Radebeul, Germany. Betaine (Cystadane powder 180 g) was from Orphan Europe, F-92800 Puteaux, France. Pegylated IFNα2b (PegIntron) and ribavirin (Rebetol 200 mg) were provided by Essex Chemie AG, Luzern, Switzerland. All study drugs were stored and distributed by the hospital pharmacy of the University Hospital Basel, Switzerland.

### Study Design

**Figure 1:**
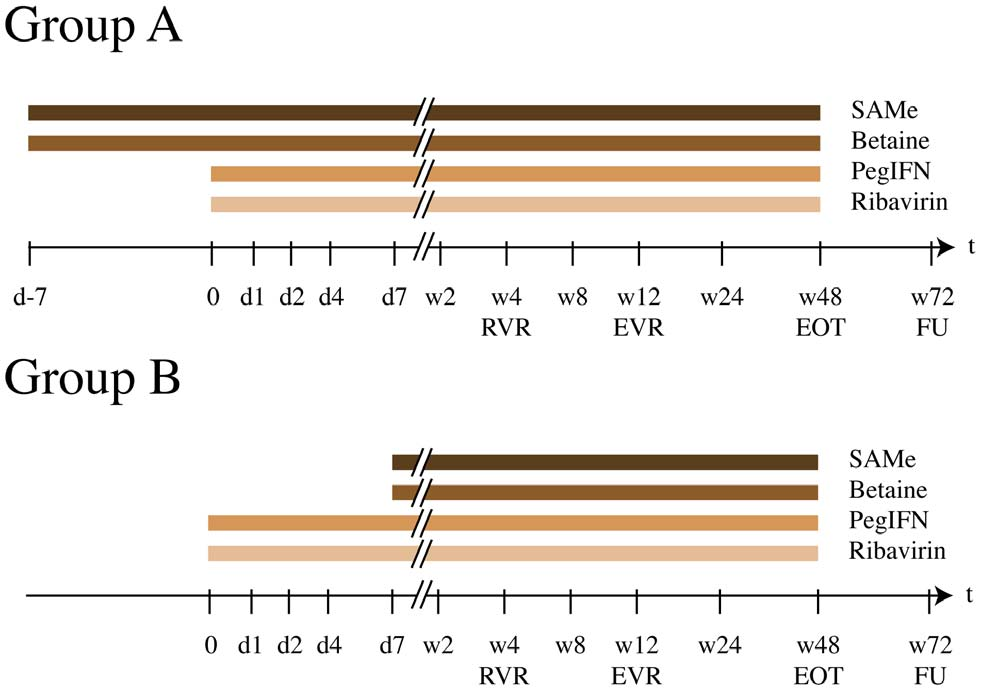
Design of the study. Patients were randomized into two groups. Patients in group A received a pre-treatment for one week with SAMe (1200 mg/day) and betaine (6 g/day), and were then treated for 3 (nonresponders) to 6 (genotypes 2 and 3) or 12 (genotypes 1 and 4) months with a combination of SAMe, betaine, ribavirin twice daily p.o. at a weight adjusted total daily dose of 800–1200 mg, and pegIFNα2b (1.5 µg/kg) injected s.c. once per week. Patients in group B received pegIFNα2b and ribavirin during the first week, followed by a combination of pegIFNα2b, ribavirin, SAMe and betaine at the same doses as outlined above for group A. The figure depicts treatment duration at the example of a patient infected with HCV genotype 1.

This single-center study was performed at the University Hospital Basel, Switzerland, Division of Gastroenterology and Hepatology. It was designed as a pilot study to test the efficacy and safety of a combination treatment with pegIFNα2b, ribavirin, SAMe and betaine in patients with CHC who did not respond to a previous therapy with (peg)IFNα and ribavirin. The primary endpoints was sustained virologic response (no HCV RNA detectable 24 weeks after the end of treatment). The secondary endpoint was early virologic response after 12 weeks of therapy with pegIFNα2b, ribavirin, SAMe and betaine. Patients were randomized into one of two treatment groups (**Figure 1**). Patients in group A received a pre-treatment with SAMe 400 mg three times daily p.o. (1200 mg/day) and betaine 3 g twice daily p.o. (6 g/day) for one week, and were then treated for 3 to 12 months with a combination of SAMe, betaine, ribavirin twice daily p.o. at a weight adjusted total daily dose of 800 mg (<65 kg body weight), 1000 mg (65–85 kg) or 1200 mg (>85 kg), and pegIFNα2b (1.5 µg/kg bodyweight) injected s.c. once per week. Patients in group B received pegIFNα2b and ribavirin during the first week, followed by a combination of pegIFNα2b, ribavirin, SAMe and betaine at the same doses as outlined above for group A. Treatment was discontinued after 12 weeks in patients who did not achieve early virological response (EVR), defined as a reduction of HCV viral load (VL) by >2 log_10_ IU/ml from baseline (BL). Duration of treatment was dependent on HCV GT and was 24 weeks for GTs 2 and 3 and 48 weeks for GTs 1 and 4. All patients had a follow-up (FU) visit 24 weeks after the end of treatment.

Patients were evaluated as outpatients for safety, tolerance, and efficacy at the start of the therapy (d-7 of group A; d0 of group B), at 24 h (d1), 48 h (d2), 96 h (d4) and 144 h (d7) after the first injection of pegIFNα2b. Further evaluations during the combination therapy were at weeks 2 (w2), w4, w8, w12, w24 and w48, and at FU (w72) 24 weeks after the end of treatment (**Figure 1**). HCV RNA was quantified with the COBAS® AmpliPrep Taqman HCV-Test from Roche Molecular Systems according to the manufacturer's instructions. HCV genotyping was performed by reverse hybridization (Inno Lipa HCV, Innogenetics, Gent, Belgium). Histopathological grading and staging of the HCV liver biopsies according to the Metavir classification system was performed at the Pathology Institute of the University Hospital Basel.

Adverse events were documented throughout the study duration.

### Ethics Statement

The study was approved by the ethics committee of the Kanton Basel and by Swissmedic and was carried out according to the Declaration of Helsinki and the International Conference on Harmonisation/Committee for Proprietary Medicinal Products (ICH/CPMP) guidelines “Good Clinical Practice”. All patients gave written informed consent before enrollment. The study conduct was monitored by KMS Kammermann Monitoring Services GmbH, Zug, Switzerland. The study was registered at ClinicalTrials.gov under the registration number NCT00310336.

## Results

### Characteristics of the Patients

**Figure 2:**
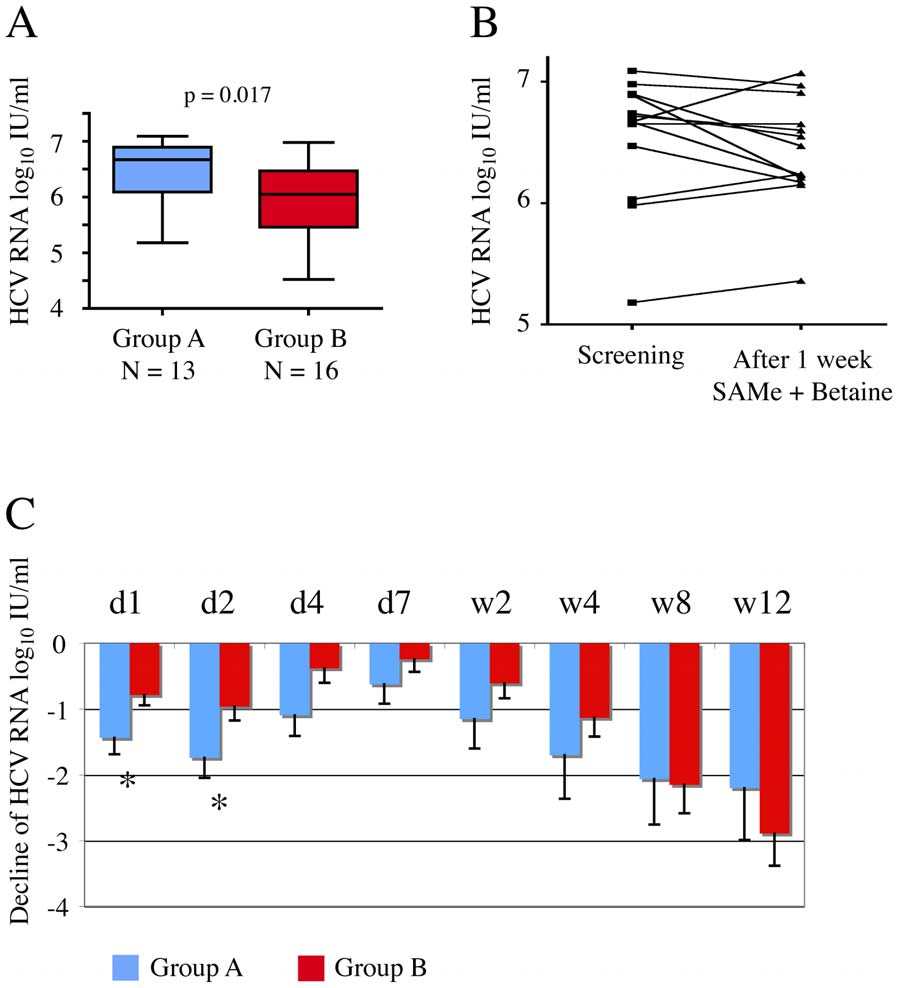
Effect of pretreatment with SAMe and betaine on HCV viral load. **A**) HCV viral load (VL) at baseline (BL) was slightly higher in group A than group B (6.51 versus 6.0 log_10_ IU/ml, t-test p = 0.017). Shown is a box plot diagram. **B**) In group A, no significant effect of SAMe and betaine pretreatment on HCV VL was observed after one week. **C**) Reduction in HCV VL compared to the BL value (in log_10_ IU/ml) during the first 12 weeks of treatment in group A (dark grey bars) and group B (light grey bars). VL reduction was significantly different at day 1 and 2, but not at later time points (unpaired t-test, p = 0.03 at d1, p = 0.04 at d2).

25 male and 8 female patients between 30 and 64 years of age, all Caucasians, were screened and enrolled in the study from October 2006 to April 2008. All patients had been previously treated in the hepatology outpatient clinic of the University Hospital of Basel. 16 patients were randomized into group A, and 17 into group B (see **Figure 1**). The randomization was performed in two separate groups for GTs 1/4 and for GTs 2/3. 2 male patients, both randomized into group A, withdrew their consent before the first application of study medication. One male patient from group A discontinued treatment after 4 days, and another male patient from group B withdrew from the study 2 weeks after treatment initiation. The remaining 29 patients were treated according to the protocol with the exception of two patients (#18 and #20) who received a liver transplant after 5 and 9 months of therapy because of development of hepatocellular carcinoma. The baseline characteristics of the 29 study patients are shown in Table 1. 23 patients were infected with HCV GT1 (79%), and 2 each with GT2 (7%), GT3 (7%) and GT4 (7%). The mean VL was 6.24 log_10_ IU/ml. 21 patients (72%) had advanced fibrosis (Metavir stages 3 or 4), 16 patients (55%) were cirrhotic. 76% of the patients had previously been treated with pegylated IFNα2a or -2b and ribavirin, whereas the remaining 24% had a previous treatment with standard IFNα2a or -2b and ribavirin (**Table 1**). Only 3 patients had had an EVR without end-of-treatment response (EoTR) to the previous treatment, whereas the majority of the patients (86%) had had less than 2 log_10_ reduction of VL within the first 12 weeks of the previous treatment (primary nonresponse, PNR). For one female patient there was no week 12 VL available from the previous treatment, she however showed an EoT nonresponse (EoTNR). Patients in group A were not significantly different from patients in group B in any of the characteristics listed in Table 1, except for VL at baseline (**Figure 2A**).

**Table 1:** Patient Characteristics.

Patient Number (male/female)	29 (21/8)
Body weight, kg (± SD)	75.83 (±15.15)
Age, years (± SD)	49.72 (±8.84)
ALAT, IU/ml (± SD)	104 (±42)
GGT, IU/ml (± SD)	184 (±161)
*HCV genotype (GT)*	
GT1, N (%)	23 (79.3%)
GT2, N (%)	2 (6.9%)
GT3, N (%)	2 (6.9%)
GT4, N (%)	2 (6.9%)
Viral load, log_10_ IU/ml, mean (range)	6.24 (4.52–7.09)
*Fibrosis stage (Metavir)*	
F1/F2, N (%)	8 (27.6%)
F3, N (%)	5 (17.2%)
F4, N (%)	16 (55.2%)
*Previous treatment*	
Pegylated IFNα/ribavirin	22 (76%)
IFNα/ribavirin	7 (24%)
*Response to previous treatment*	
Primary nonresponse	25 (86%)
Early virological response but no end-of-treatment response	3 (10%)
Week 12 response unknown, but no end-of-treatment response	1 (3%)

### Virological and Biochemical Response to SAMe and Betaine

To test whether SAMe and betaine have an effect on VL and liver enzyme values that is independent from pegIFNα and ribavirin, patients in group A were pre-treated for one week with SAMe and betaine only. There was no significant decrease in VL in response to SAMe and betaine (**Figure 2B**), and no significant effect on ALAT serum levels (data not shown). The mean reduction of VL for both treatment groups at days 1, 2, 4, and 7 and at weeks 2, 4, 8 and 12 is shown in **Figure 2C**. Group A (pretreatment with SAMe and betaine) had a slightly more pronounced reduction of VL in the first 48 h of combination treatment (unpaired t-test; p = 0.03 for d1; p = 0.04 for d2), there was however no statistically significant difference between the two groups (A and B) at later time points, and even the significant results at early time points have to be interpreted with caution, given the fact that baseline VL was higher in group A.

### Virological and Biochemical Response to Treatment with PegIFNα2b, Ribavirin, SAMe and Betaine

**Figure 3:**
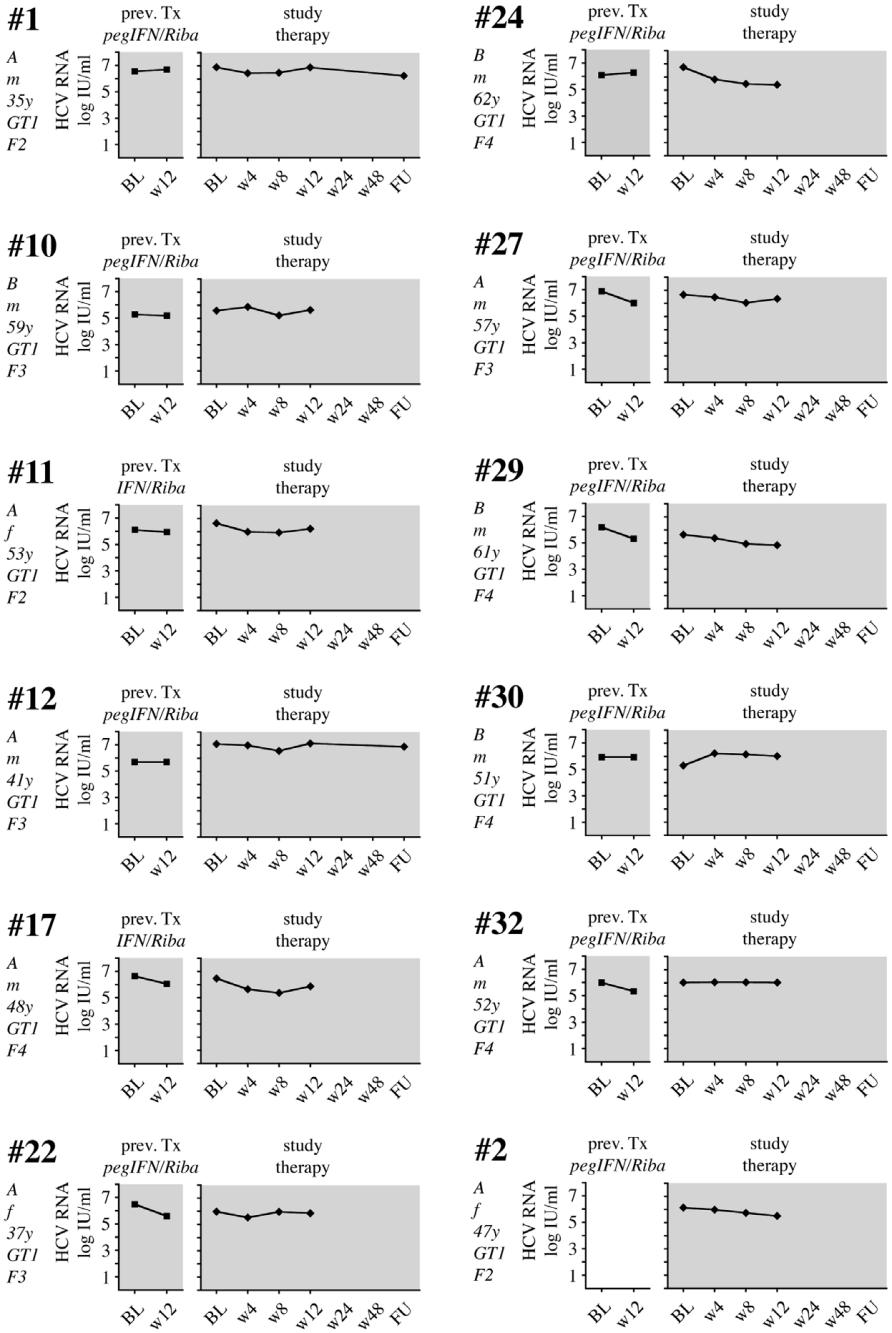
Viral load in 12 patients with nonresponse to pegIFNα2b, ribavirin, SAMe and betaine. 12 patients had no virological response to the study medication (Primary non-response, PNR, color code  =  grey). The left panel shows VL during the first 12 weeks of the previous treatment. The right panel shows VL during the study therapy. Indicated on the left from top to bottom are treatment group (A or B), sex (m or f), age at the start of the therapy, GT and Metavir fibrosis stage. The previous treatment (either pegIFNα/ribavirin oder IFNα/ribavirin) is indicated on top of the left panel.

**Figure 4:**
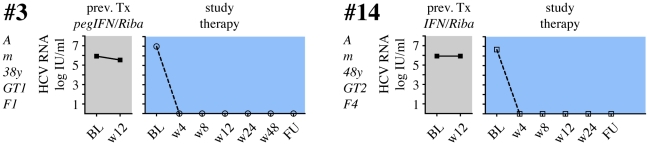
Viral load in 2 patients with rapid virological response to pegIFNα2b, ribavirin, SAMe and betaine. 2 patients showed an RVR to the study treatment (color code  =  blue), both had no virological response in the previous treatment. Both patients had an SVR. The left panel shows VL during the first 12 weeks of the previous treatment. The right panel shows VL during the study therapy. Indicated on the left from top to bottom are treatment group (A or B), sex (m or f), age at the start of the therapy, GT and Metavir fibrosis stage. The previous treatment (either pegIFNα/ribavirin oder IFNα/ribavirin) is indicated on top of the left panel.

**Figure 5:**
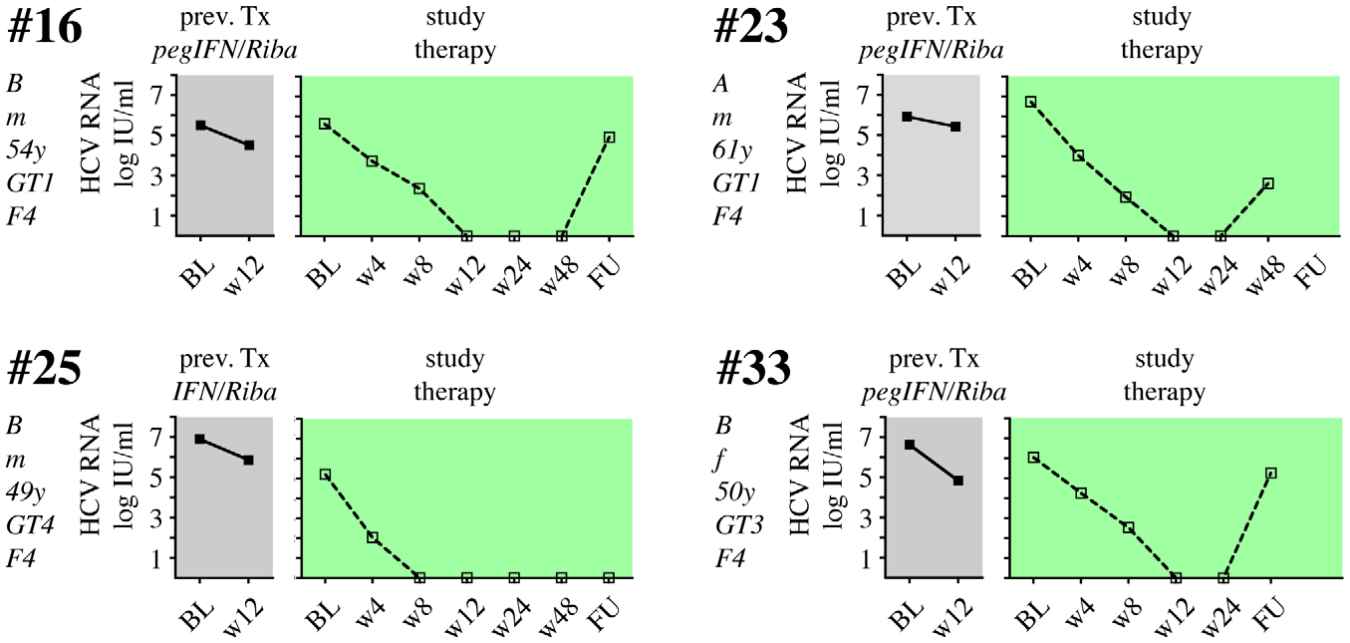
Viral load in 4 patients with a complete early virological response to pegIFNα2b, ribavirin, SAMe and betaine. 4 patients with a cEVR to the study treatment (color code  =  green), all of them previous nonresponders. Patient #25 had an SVR.

**Figure 6:**
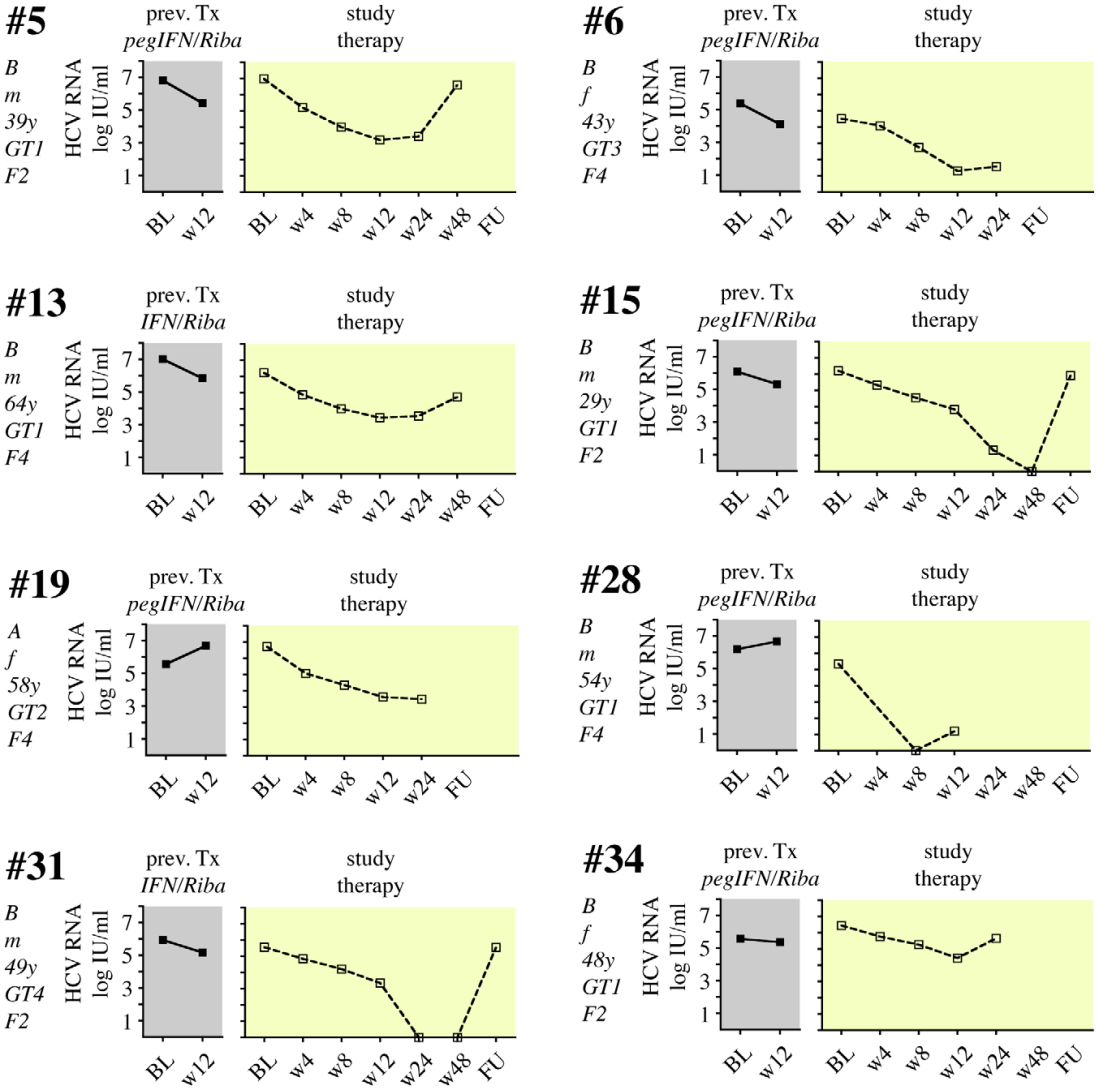
Viral load in 8 patients with an early virological response to pegIFNα2b, ribavirin, SAMe and betaine. 8 patients had >2 log reduction of VL after 12 weeks of combination treatment but had still detectable HCV RNA (EVR, color code  =  yellow). All of them had been previous nonresponders. No patient in this group had an SVR.

**Figure 7:**
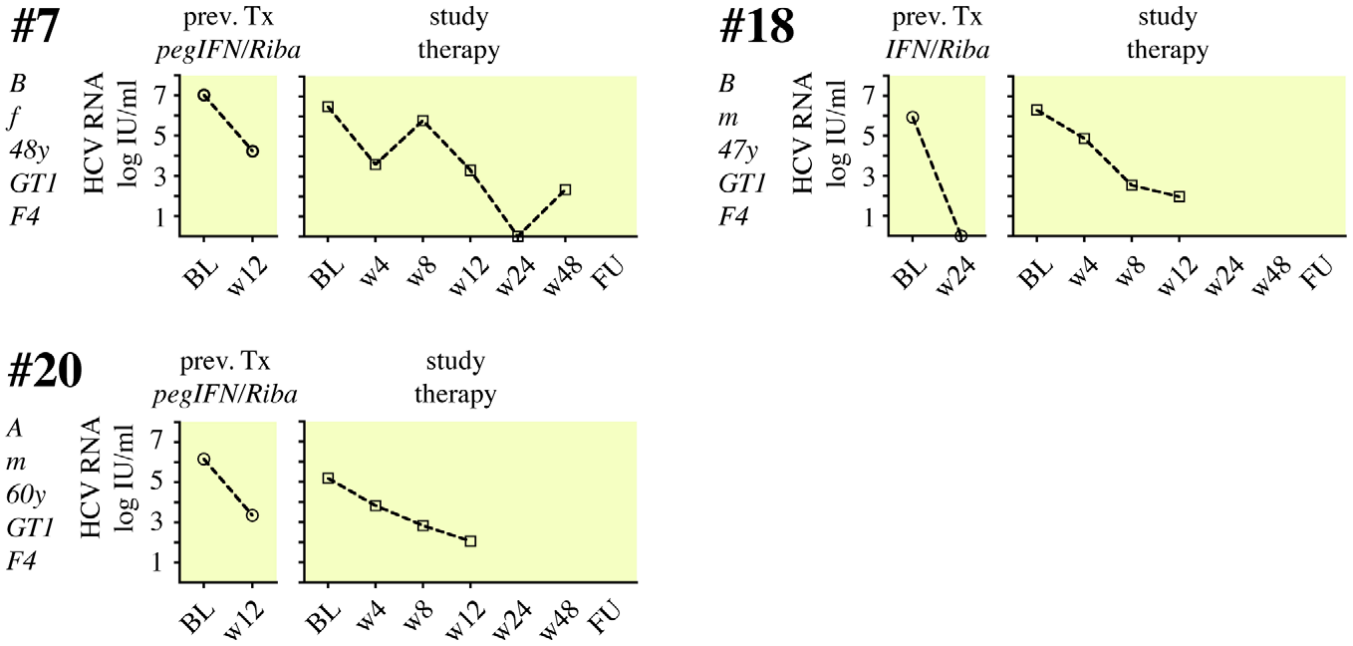
Viral load in response to pegIFNα2b, ribavirin, SAMe and betaine in 3 patients with an EVR in their previous treatment. 3 patients had achieved an EVR in their previous treatment, but no EoTR. They all achieved EVR when re-treated with the study medication. None of them had an SVR.

Twelve patients (41%) did not achieve an EVR with the study combination treatment (**Figure 3**), and therapy was discontinued after 12 weeks. The remaining 17 patients (59%) showed an initial virological response, although 14 formerly had a documented PNR in their previous treatment. Two patients (patients #3 and #14) now showed rapid virological response (RVR) with undetectable HCV-RNA after 4 weeks of the study treatment, and both had SVR in follow-up (**Figure 4**). Four patients had negative HCV RNA after 12 weeks of study treatment (complete EVR; cEVR), and one of them (#25) completed study treatment with a SVR (**Figure 5**). The other 3 patients relapsed either on treatment (#23) or during follow-up (#16, #33). All 4 cEVR patients were cirrhotic, and 3 of them had been previously treated with pegIFNα2 and ribavirin (**Figure 5**). The remaining eight patients with previous PNR had an EVR at week 12 of study combination therapy (**Figure 6**). However, only two of them became HCV RNA negative on treatment, and none of them had an SVR. Finally, there were three patients who had achieved an EVR but no EoTR in their previous treatments with (peg)IFNα2 and ribavirin (**Figure 7**). All of them again showed an EVR. Study treatment had to be discontinued however in patients #18 and #20 because they were liver transplanted during the study (at months 5 and 9 of treatment, respectively).

Hence, 15 patients with an initial response (either RVR or cEVR or EVR) to the study medication completed the treatment according to the study protocol. All together, 3 of these 15 patients had an SVR, 4 had an EoTR but relapsed during FU, and 8 had no EoTR.

**Figure 8:**
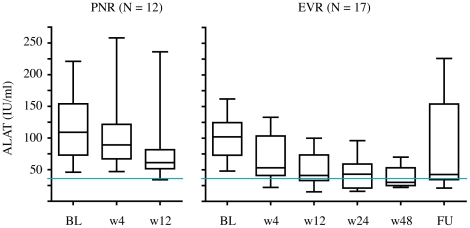
Biochemical response to study medication. Shown are box plot diagrams of the ALAT values at baseline (BL), week 4 (w4), w12, w24, w48 and follow up (FU) in 12 patients without response to the study medication (left panel) and 17 patients with an EVR (right panel). The blue line shows the upper limit of normal.

The biochemical response in the patients with an initial response to pegIFNα2b, ribavirin, SAMe and betaine combination therapy was more pronounced than in the PNR patients (**Figure 8**). At week 12 of the treatment, only 1 of the 12 PNR patients, but 7 of the 17 patients with an EVR had normal ALAT.

### Adverse Events and Dose Modification

The patients reported side effects such as flu-like symptoms (17 patients), Fatigue (11), myalgias and arthralgias (11), dry skin (10), moodiness and depression (9), diarrhea (8), chills and shivering (8), fever (6), abdominal pain and cramps (6), headache (6), loss of appetite (4), nausea (3), dizziness (3), exertional dyspnea (3), alopezia (2), cough (1) and sleep disturbances (1), that did not differ from the ones usually observed in pegIFNα and ribavirin treatments. No unexpected or unusual adverse events occurred during the entire study. No adverse events occurred during the first 7 days of treatment with SAMe and betaine in patients from group A. Hemoglobin, platelets and neutrophils decreased during therapy as is expected for pegIFNα and ribavirin combination treatments. The pegIFNα dosage was reduced in 4 patients (by 20 µg/week in patients #3, #5 and #7, and by 38 µg/week in patient #23). The ribavirin dosage was reduced in 2 patients, from 1000 mg/day to 800 mg/day (patient #13) and from 1200 mg/day to 1000 mg/day (patient #16).

## Discussion

**Figure 9:**
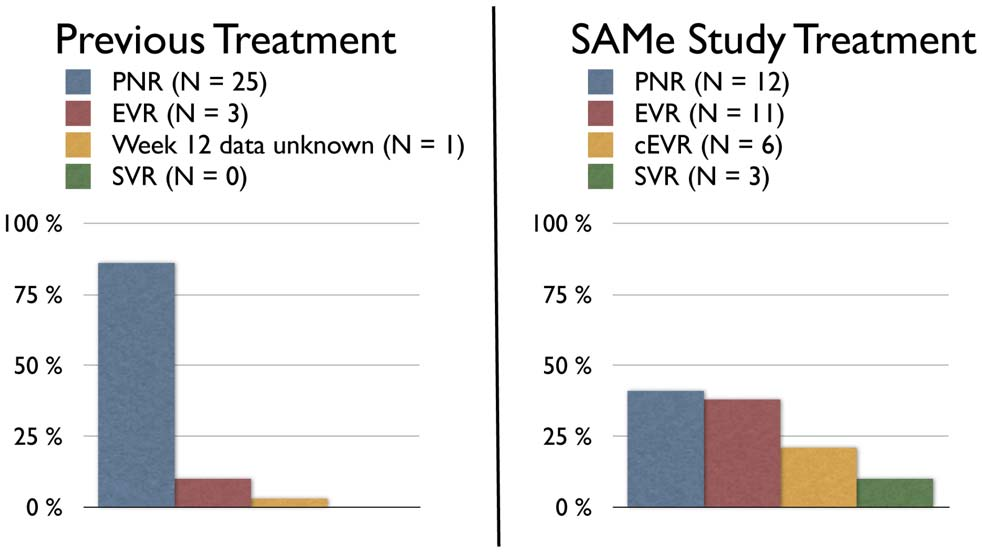
Distribution of early virological response to previous treatments and to the study medication. 86% of the 29 study patients had a primary non-response (PNR) to their previous combination treatments with (peg)IFNα/ribarivin. The rate of PNR was reduced to 41% by addition of SAMe and betaine to pegIFNα/ribavirin. Three patients achieved an SVR with the study medication.

This pilot trial included 29 patients who had previously failed a combination therapy with (peg)IFNα and ribavirin. 17 (59%) of these patients now had an EVR when re-treated with pegIFNα2b, ribavirin, SAMe and betaine (**Figure 9**). The addition of SAMe and betaine to the current standard combination therapy therefore achieved an EVR rate that was considerably higher than reported before in trials investigating re-treatment of patients with previous nonresponse and relapse. The recently published EPIC trial, investigating response to pegIFNα2b/ribavirin therapy in 2293 patients with previous (peg)IFNα/ribavirin therapy failure, showed that overall 36% of the patients achieved an EVR [14]. EPIC, importantly, not only included nonresponders (61%), but also at least 28% relapse patients. This is important because re-treatment of relapse patients has consistently resulted in higher SVR rates than re-treatment of nonresponders [14], [15], [16], [17], [18]. Moreover, 37% of the patients included in EPIC had previously been treated with standard IFNα/ribavirin. In our trial we only included nonresponders, 86% of which even had documented PNR at week 12 of the previous treatment, and 76% had been treated with pegIFNα/ribavirin (only 24% with IFNα/ribavirin).

Taken together, the 59% EVR rate in our difficult-to-treat group of patients suggests that the addition of SAMe and betaine to pegIFNα2b/ribavirin improves EVR rates in patients with CHC.

Despite the high rate of EVR in our trial, only 3 patients (10%) achieved an SVR. 4 additional patients achieved an EoTR but then relapsed during FU. 10 of the 17 EVR patients, however, either never reached negativity for HCV RNA or had a viral breakthrough during therapy. Overall, only 17% of the patients with an EVR showed later SVR in our study. Other re-treatment studies reported higher SVR rates (49% [15]; 36% [16]) in patients who had an EVR during re-treatment with pegIFNα/ribavirin. In EPIC, an SVR was observed in 56% of the patients who were HCV RNA negative at week 12 (cEVR), in 12% of the patients with an EVR but still detectable HCV-RNA at week 12, and in none of the patients without EVR [14]. In our small pilot study, 50% (3 of 6) of patients with a cEVR had an SVR, but none of the patients with an EVR (and still detectable HCV RNA at week 12). Because of the heterogeneity of these clinical trials it is difficult to directly compare the results. However, the overall low SVR rate in our EVR patients suggests that SAMe and betaine are only effective early in the treatment. It is conceivable that adaptive mechanisms in hepatocytes restore intracellular SAMe concentrations to pre-treatment levels, because SAMe is involved in many enzymatic reactions and its concentration is regulated by numerous metabolic pathways.

Tachyphylaxis to SAMe might be overcome by increasing the dosage, but in this small pilot study, we were not able to evaluate the effectiveness of different doses of SAMe and betaine. SAMe was given at a dose of 1200 mg per day because the same dosage was used and found to be safe during a treatment period of two years in the largest study published using SAMe [19]. Likewise, betaine treatment with 6 g per day has been previously shown to be effective and safe [20]. Future studies should aim to define the optimal dosages for SAMe and betaine.

The rationale for adding SAMe to pegIFNα/ribavirin treatments is based on our previous analysis of HCV interference with IFNα signaling in cultured cells, transgenic mice and liver biopsies from patients with CHC [6], [8], [9], [13], [21]. Using cell lines with inducible expression of HCV proteins and using the HCV replicon system we showed that SAMe improves methylation of the IFNα induced transcription factor STAT1 and its binding to DNA response elements, thereby increasing the induction of ISGs and enhancing the inhibitory effect of IFNα on replicons [13]. In the present clinical study we could not assess the effects of SAMe and betaine on IFNα signal transduction in the liver of the patients. Consequently, we cannot exclude that SAMe and betaine improve the response to pegIFNα/ribavirin treatments by mechanisms that differ from our proposed model of action that involves methylation of STAT1 as the key event.

Betaine was added to the combination treatment in order to prevent the accumulation of the toxic SAMe metabolite S-adenosyl-L-homocysteine (AdoHcy) and to further increase the intracellular concentrations of SAMe [12], [22]. We did not measure the plasma levels of homocysteine or betaine, and therefore cannot assess the effect of betaine on SAMe metabolism in our patients.

A number of directly acting antiviral agents is presently in advanced stages of clinical development. The most promising candidates include direct inhibitors of the HCV NS3 protease such as telaprevir [23], boceprevir [24] and MK-7009 [25], as well as both nucleoside and non-nucleoside inhibitors of the NS5B RNA-dependent RNA polymerase. Although these agents have demonstrated potent antiviral effectiveness, monotherapy has been complicated by rapid virological breakthrough due to the selection of drug-resistant mutants. To prevent viral resistance, protease and polymerase inhibitors are used only in combination with pegIFNα/ribavirin in phase II and III clinical studies. However, a substantial number of patients do not respond to pegIFNα, most likely because their endogenous IFN system is already induced before therapy, and because of HCV interference with IFNα signal transduction through the Jak-STAT pathway [6], [8], [21], [26], [27], [28]. Such patients might be at risk for viral breakthrough and resistance development even when receiving a triple combination therapy (protease inhibitor plus pegIFNα/ribavirin). The 4 weeks of lead-in phase with pegIFNα/ribavirin used in SPRINT-1 and in ongoing boceprevir trials will exclude those flat nonresponders from the triple combination therapy, and will help to reduce the emergence of resistant viruses [24]. The addition of SAMe and betaine to the initial phase of such a treatment could be a reasonable strategy. By improving the cellular response to pegIFNα, SAMe and betaine have the potential to increase the number of patients who achieve an initial response and can be offered triple combination therapies.

In conclusion, addition of SAMe and betaine to the current standard therapy with pegIFNα/ribavirin resulted in an EVR in 59% of patients who previously were nonresponders to (peg)IFNα/ribavirin therapies. SAMe and betaine had an excellent safety and tolerability profile. The improvement of response to pegIFNα/ribavirin, when combined with SAMe and betaine, should encourage further studies, since pegIFNα and ribavirin will be cornerstones of CHC treatments for many years to come.

## Supporting Information

Checklist S1 CONSORT Checklist (DOC)

Protocol S1 Trial Protocol (PDF)
