# Correction: S-Adenosyl-Methionine and Betaine Improve Early Virological Response in Chronic Hepatitis C Patients with Previous Nonresponse

**DOI:** 10.1371/annotation/1e4a3fa2-2189-441f-809c-4fbf077e34e8

**Published:** 2010-11-17

**Authors:** Magdalena Filipowicz, Christine Bernsmeier, Luigi Terracciano, Francois H. T. Duong, Markus H. Heim

The first two authors, Magdalena Filipowicz and Christine Bernsmeier, should be noted as contributing equally to this work. 

