# Supplementary material for: S-Adenosyl-Methionine and Betaine Improve Early Virological Response in Chronic Hepatitis C Patients with Previous Nonresponse
Source: PLoS One. 2010 Nov 8;5(11):e15492. doi: 10.1371/journal.pone.0015492 (PMC2975710; doi:10.1371/journal.pone.0015492)
Supplement: Protocol S1 — Trial Protocol (PDF) [file pone.0015492.s002.pdf]

Study Protocol

Title:
Chronic Hepatitis C: Treatment of (peg)IFNα-Ribavirin Non-Responders with Pegylated Interferon α2b (PegIntron), Ribavirin, AdoMet and Betaine

Principle Investigator:
Prof. Markus Heim
University Hospital Basel
Division of Gastroenterology and Hepatology

Co-Investigators:
Dr. Christine Bernsmeier, MD
University Hospital Basel
Division of Gastroenterology and Hepatology

Dr. Magdalena Filipowicz, MD
University Hospital Basel
Division of Gastroenterology and Hepatology

1. Background

The current treatment of chronic hepatitis C (CHC) with pegylated interferons plus ribavirin achieves a sustained response in less than 50% of all patients (1, 2). Over the last 10 years, several research groups including our own studied the apparent interferon resistance caused by hepatitis C virus (HCV). These studies have revealed an amazing ability of HCV to interfere with and inhibit the innate immunity, specifically the interferon system. Type I interferons (IFNαs and IFNβ) are induced upon viral infection through the activation of a pathway involving RIG-I, TBK1, IKKε, IRF3 and NFκB (3-9). They are then secreted and can bind to the IFNα receptor (IFNAR) on the same cell (autocrine amplification loop) or on neighboring cells (paracrine amplification loop), or can be transported through the blood circulation to distant sites. IFN binding to IFNAR activates the Jak-STAT signal transduction pathway (10, 11). Activated STAT1 (signal transducer and activator of transcription) and STAT2 stimulate the transcription of several hundreds of ISGs (IFN stimulated genes). Amongst them are genes that are important to establish an antiviral state in the cells, but also additional IFNα genes, and IRF7, an important transcription factor for the activation of IFNα genes. This positive feedback loop results in a strong antiviral response by the host.

HCV targets this system at three levels. First, the viral non structural protein 3/4 (NS3/4) inhibits RIG-I (12). Second, NS3/4 proteolytically cleaves TRIF, an adaptor protein linking Toll-like receptor 3 to IFN gene induction (13). In both cases, the induction of IFN expression by HCV infected cells is blocked. Third, HCV interferes with IFN induced signaling through the Jak-STAT pathway. Our own research group has focused their studies on this third mechanism of HCV interference with the IFN system. A key finding, published in the Journal of Virology 1999, was the observation that interferon induced activation and binding of ISGF3 and of STAT1 dimers is inhibited in cells that express HCV proteins (14). ISGF3 (interferon stimulated gene factor 3) and STAT1 (signal transducer and activator of transcription 1) are essential for interferon signal transduction. Both mice and human patients that lack STAT1 have a severe impairment of the interferon system and susceptible to (viral) infections (15-17).

A detailed analysis of the molecular mechanisms responsible for the inhibition of Jak-STAT signaling by HCV revealed a novel mechanism of viral interference with IFN signaling. HCV infection induces the expression of protein phosphatase 2A (PP2A) (18). PP2A is a direct inhibitor of protein arginine methyltransferase 1 (PRMT1) (19), PRMT1 is responsible for arginine methylation of STAT1, an important posttranslational modification that controls the association of STAT1 with its inhibitor PIAS1 (protein inhibitor of activated STAT1)(20). Therefore, by inducing the expression of PP2A, HCV indirectly induces the binding of PIAS1 to STAT1 (Figure 1). As a consequence, ISGs that are susceptible to inhibition of STAT1 by PIAS1 are not induced (19, 21).

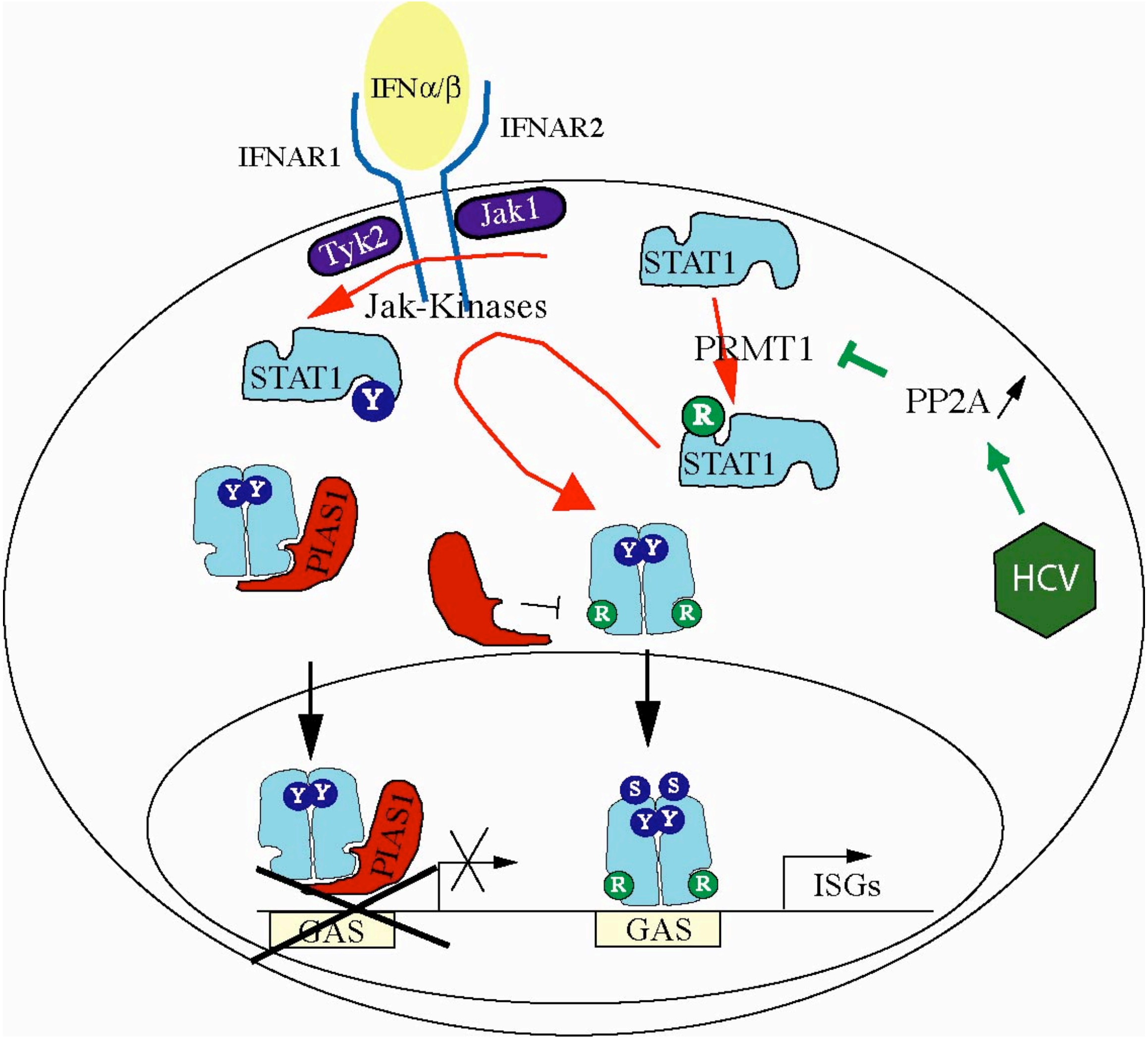

Figure1:
Binding of STAT1 to response elements in the promotors of IFN stimulated genes (ISG) is controlled by PIAS1 (protein inhibitor of activated STAT1). PIAS1 can bind to unmethylated STAT1 dimers (left side of figure), but not to methylated STAT1 dimers (right side of figure). HCV inhibits the methylation of STAT1 by inducing PP2A, a direct inhibitor of PRMT1.

The reaction equilibrium of PRMT1 can be driven to the product side (methylated substrates, for example methylated STAT1) by increasing the concentration of the substrates. S-Adenosyl-L-Methionine (AdoMet) is the methyl group donor in the reaction. Its concentration can be increased by adding AdoMet to the reaction in vitro or by adding AdoMet to the culture medium of cells. AdoMet is recycled in a cycle that uses betaine as a substrate for betaine homocysteine methyltransferase (Figure 2), and the concentration of AdoMet can also be increased by adding betaine.

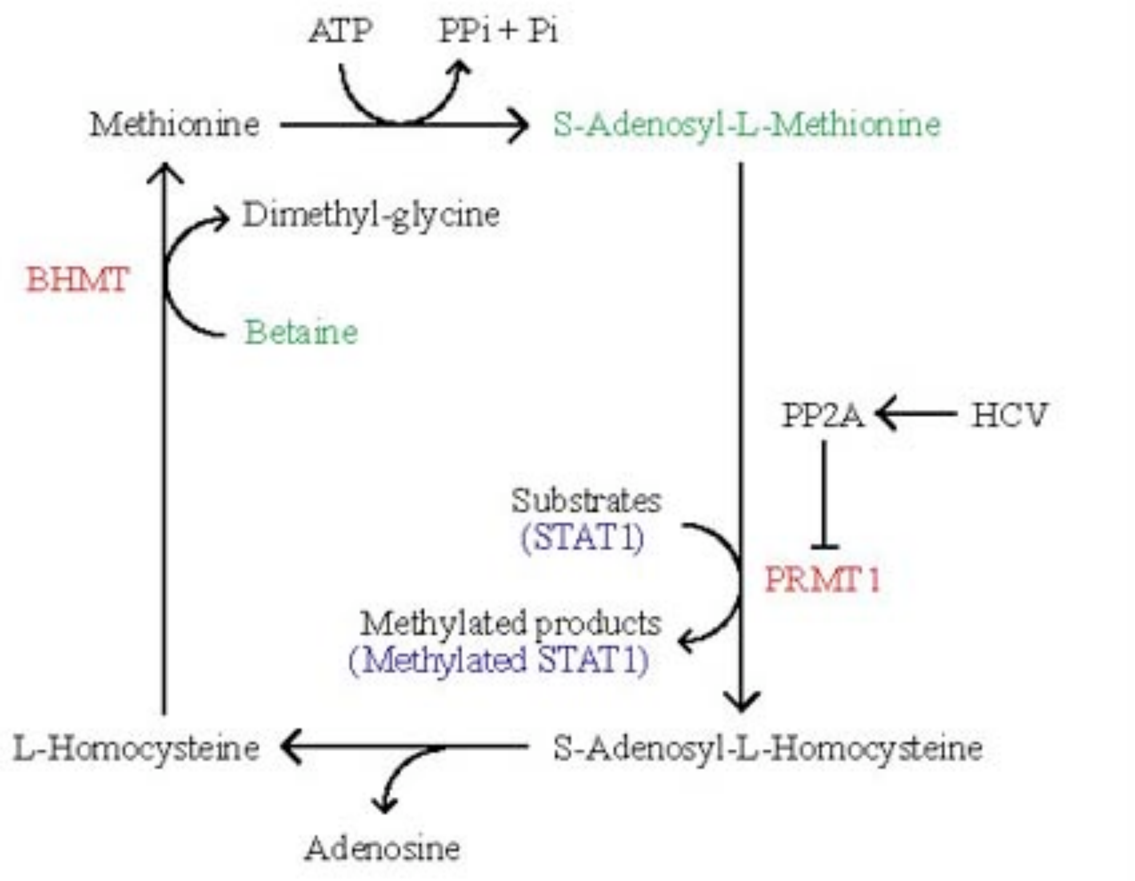

Figure 2:
S-Adenosyl-L-Methionine is the methyl group donor in methylation reactions catalysed by PRMT1 (for example methylation of STAT1). S-Adenosyl-L-Methionine is recycled from L-Homocysteine through Methionine. Betaine is a substrate of Betaine-Homocysteine-Methyltransferase.

In as yet unpublished work, we found that the treatment of cells with the methyl group donors S-adenosylmethionine (AdoMet) and betaine restores the sensitivity to IFNα in cells that express HCV proteins. Furthermore, in Huh7 cells harboring a HCV replicon, the inhibitory effect of IFNα on the replicon was ten times stronger when the cells were treated with IFNα plus AdoMet and betaine. Both AdoMet and betaine have been used in human for many years, and are over the counter drugs in many countries (not Switzerland). They are used for a number of indications such as alcoholic liver disease, non-alcoholic liver disease or hyperhomocysteinemia. They are taken orally, and have an excellent safety record.

Based on our result using biochemical assays, cell culture assays with cells expressing HCV proteins, and Huh7 cells harboring a HCV replicon we hypothesize that adding AdoMet and betaine to the current standard therapy with pegylated IFNα plus ribavirin can enhance the efficacy of the treatment, and will result in sustained responses even in patients that have been non-responders or relapser to (peg-)IFNα plus ribavirin treatments.

2. Rational of the Study

- 50-60% of patients with chronic hepatitis C are not cured by treatment with pegylated IFNα plus ribavirin.
- Retreatment of non-responders of previous (pegylated) IFNα plus ribavirin therapies with pegylated IFNα plus ribavirin results in a sustained response in less than 10% of the patients.
- Extensive analysis of IFNα signaling in cells expressing HCV proteins, in transgenic mice expressing HCV proteins, and in liver biopsies from patients with chronic hepatitis C point to STAT1 methylation as an important posttranslational modification targeted by HCV to inhibit IFNα signaling.
- STAT1 methylation can be increased and IFNα can be improved by adding AdoMet and betaine.
- Analysis of mRNA expression levels of interferon stimulated genes (ISGs) in PBMCs (RNA extracted from whole blood) can be used as a surrogate marker for the activation of the IFN system.

3. Objectives of the Study

The study is designed to test the hypothesis that a combination treatment with pegylated IFNα2b, ribavirin, AdoMet and betaine is superior to the current standard combination therapy with pegylated IFNα plus ribavirin.

4. Experimental Design

Prospective, randomized, open label pilot study to test the efficacy and safety of a combination treatment with PegIntron, Rebetol, AdoMet and betaine in
non-responders
of previous combinations therapy with (peg)IFNα and Ribavirin.

Half of the patients (Group A) will be treated during the first week with AdoMet and Betaine, followed by a combination treatment of PegIntron, Rebetol, AdoMet and Betaine.
The other half of the patients (Group B) will be treated during the first 1 week with PegIntron and Rebetol, followed by a combination treatment of PegIntron, Rebetol, AdoMet and Betaine. (This randomized assignment to groups A or B will allow to test the usefulness of the surrogate marker ISG induction for predicting treatment response). The randomization will be done in two separate groups for genotype 1 and 4 and for genotypes 2 and 3, respectively. In each group, a list with a randomized sequence of A's and B's in blocks of 10 will be used to assign the patients into groups A or B.

The study will be performed at the University Hospital Basel.

5. Patient Selection

Number of patients:
30 patients, non-responders to previous (peg-)IFNα - Ribavirin combination therapy

Inclusion criteria:

- Male and female between 18 and 65 years.
- Non-responders in previous treatments with IFNα plus ribavirin or pegylated IFNα plus ribavirin.
- Elevated ALT-levels on at least two occasions during ≥6 months preceding entry.
- Detection of HCV RNA in serum (PCR).
- Compensated liver disease (Child-Pugh A) and a Child-Pugh score ≤5.
- The following minimal hematologic and biochemical criteria:
- Hemoglobin for males and females ≥11g/dl
- Absolute Neutrophil count ≥1500 cells/mm^3
- Platelets ≥75'000/mm^3
- HBs Ag negative.
- ANA ≤1:320, and no evidence for autoimmune hepatitis.
- α-Fetoprotein ≤50μg/l (when between upper limit of normal and 50μg/l, ultrasonographical exclusion of hepatocellular carcinoma (HCC) is needed).
- Fasting blood glucose within normal limits, if history of diabetes or hypertension, a pre-therapy ocular examination is indicated.
- TSH within normal limits or adequately controlled.
- Negative urine or blood pregnancy test (for women of childbearing potential) documented within the 2-3 week period prior to the first dose of study drug. Additionally, all fertile males and females must be using effective contraception during treatment and during the 6 months after treatment end. This may include, but is not limited to, using birth control pills, IUDs, condoms, diaphragms, or implants, being surgically sterilized, or being in a post-menopausal state.
- Willingness to give written informed consent and willingness to participate to and comply with the study.

Exclusion criteria:

- Women with ongoing pregnancy or breast feeding.
- Positive test at screening for anti-HAV IgM Ab, HBsAg, anti-HBc IgM Ab, HBe Ag.
- Positive test at screening for HIV.
- History or other evidence of a medical condition associated with chronic liver disease other than HCV (e.g., hemochromatosis, autoimmune hepatitis, alcoholic liver disease, toxin exposures).
- Hypersensitivity to study drugs.
- Participation in any other clinical trial within 30 days of entry into this protocol.
- Treatment with any investigational drug within 30 days of entry into this protocol.

- History or evidence of decompensated liver disease (Child-Pugh B/C) and a Child-Pugh score >5. Ascites, coagulopathy, hyperbilirubinemia, hepatic encephalopathy, or hypoalbuminemia and a Child-Pugh score >5 are conditions consistent with decompensated liver disease.
- History or other evidence of bleeding from esophageal varices or other conditions consistent with decompensated liver disease.
- Hepatocellular carcinoma (HCC) or α-Fetoprotein >50μg/l.
- Patients with organ transplants other than cornea and hair transplant.
- Therapy with any antisystemic or immunmodulatory treatment (including supra-physiologic doses of steroids or radiation) ≤6 months prior the first dose of study drug
- Hemoglobinopathy (e.g. thalassemia) or any other cause of or tendency for hemolysis.
- Any known preexisting medical condition that could interfere with the patient's participation in and completion of the study such as:
- Preexisting psychiatric condition, especially depression, or a history of severe psychiatric disorder, such as major psychosis, suicidal ideation and/or suicidal attempts (based on a mandatory psychiatric advice).
- CNS trauma or active seizure disorders requiring medication.
- Significant cardiovascular dysfunction.
- Poorly controlled diabetes mellitus.
- Renal dysfunction, i.e. serum creatinine levels ≥1.5 times upper limit of normal.
- Autoimmune diseases.
- Evidence of severe retinopathy (e.g. CMV retinitis, macular degeneration).
- Any medical condition requiring, or likely to require during the course of the study, chronic systemic administration of steroids.
- Clinical gout.
- Important substance abuse (alcohol >80 g/d, i.v. drugs etc.).
- Active opportunistic infections.
- Non-Hodgkin lymphoma or Hodgkin lymphoma.
- Kaposi sarcoma.
- Inability or unwillingness to provide informed consent or abide by the requirements of the study.
- Male partners of pregnant women.

6. Endpoints

Primary endpoints:
1. Sustained virologic response (no HCV RNA detectable 24 weeks after the end of treatment).

Secondary endpoints:
1. Early virologic response after 12 weeks of therapy with PegIntron, Rebetol, AdoMet and betaine.
2. Cmax and AUC of mRNA induction of IFN stimulated genes over 96 hours after first injection of PegIntron (RNA extracted from whole blood).

7. Evaluation, Statistics

7.1. Primary Endpoints:

The sustained response rate in this group of patients will be compared to response rates published in other studies (=historical control). In a recent publication from the HALT-C trial, the sustained response rate of a treatment with PegIFNα2a plus ribavirin of 385 patients that were non-responders or relapser of a previous treatment with IFNα plus Ribavirin was 12% (22).

We have no preliminary data that would allow us to estimate the sustained response rate in previous (peg)IFNα plus ribavirin non-responders when treated with a combination of pegylated IFNα, ribavirin, AdoMet and betaine. We postulate that an improvement of the sustained response rate of 20% or more would be clinically relevant.
In order to detect a difference of 20% with a 5% significance (α) and 80% power (β), we estimate the sample size with the following formula:

$$n = \left[\frac{z_\alpha \sqrt{\pi_0(1-\pi_0)} - z_\beta \sqrt{\pi_1(1-\pi_1)}}{\pi_0 - \pi_1}\right]^2$$

$\pi_0 = 0.12$
$\pi_1 = 0.32$
$\alpha = 0.05$
z_α= 1.96 (two-tailed)
$\beta = 0.2$
z_β = - 0.84 (one-tailed)

$$n = \left[\frac{1.96\sqrt{0.12(1-0.12)} - (-0.84)\sqrt{0.32(1-0.32)}}{0.12-0.32}\right]^2 = \left[\frac{1.96\sqrt{0.12 * 0.88} + 0.84\sqrt{0.32 * 0.68}}{-0.2}\right]^2 = \left[\frac{1.96\sqrt{0.1056} + 0.84\sqrt{0.2176}}{-0.2}\right]^2 = 26.4$$

For this non-responder group, the sample size N = 26.4

7.2. Secondary Endpoints:

For secondary endpoint 1, virological response after 12 weeks will be assessed, and compared to a historical control, where an early virological response rates of 28% was found (22).

For secondary endpoint 2, RNA samples extracted from whole blood at baseline, and 24, 48 and 96 hours after the first injection of PegIntron will be analyzed by real-time RT-PCR (Taqman) for the concentrations of ISGs including IP-10, ISG15, ISG54, PKR and 2'5'OAS. The results from groups A and B will be compared using the Wilcoxon rank test.

8. Randomization

After inclusion in the study, the patients are randomly assigned to Group A or Group B. The randomization will be done in two separate groups for genotype 1 and 4 and for genotypes 2 and 3, respectively. In each group, a list with a randomized sequence of A's and B's in blocks of 10 will be used to sequentially assign the patients into groups A or B (appendix 4).

9. Patient Discontinuation Criteria:

It is the right and duty of the investigator to interrupt the treatment of any subject whose health or well-being may be threatened by continuation in this study, or who may be experiencing unmanageable factors that may interfere significantly with the study procedures and/or the interpretation of study results. Such subjects should be withdrawn from the study, not continued under a modified regimen.

9.1 Subject Discontinuation Criteria

Subject's participation may be terminated for any of the following reasons:

- Serious or life-threatening adverse event (AE).
- Failure to comply with the dosing schedule, study procedures, or other study requirements.
- Subject's request (subjects may discontinue treatment at any time for any reason).
- Pregnancy during the study period.
- The investigator feels that it is in the subject's best interest to discontinue treatment at that time.
- Confirmation that the subject meets the protocol's exclusion criteria with regard to history of psychosis, suicidal ideation, or suicide attempts at any time during the study.

9.2 Procedures for Discontinuation

If a subject withdraws or is removed from the study for any reason prior to the completion of the study, the reason for and date of the discontinuation and date of the last dose of study medication must be recorded in the appropriate section of the Case Report Form (CRF).

At the time of discontinuation, every effort should be made :

- to ensure all procedures and evaluations scheduled for the final treatment visit are performed (see Study Flow Chart), including the assessment of adverse events and medication compliance. Any returned drug must be inventoried.
- to make an appointment for the follow-up visit(s).

10. Study Medication and Treatment:

10.1. Supply and Drug Dispensing

10.1.1. AdoMet and Betaine

AdoMet (Samyr ®)and Betaine (Cystadane ®) will be supplied by the Pharmacy of the University Hospital Basel.

10.1.2. PegIntron and Rebetol

PegIntron® and Rebetol® will be supplied by Essex Chemie AG. Essex Chemie AG will be informed by fax of the inclusion of a patient, and will then send the supply of PegIntron and Rebetol for 6 months to the study personal of the Division of Gastroenterology and Hepatology of the University Hospital Basel. For patients that have a full 12 month treatment, a second order for PegIntron and Rebetol will be faxed to Essex Chemie AG.
Unused medication will be given back to Essex Chemie AG.

10.2. Treatment of Group A Patients:

Dosage (dose adjustment guidelines: see Appendix 3): patients included will receive one week of pretreatment with:

1. AdoMet 3 x 400mg/d p.o.
2. Betaine 2 x 3g/d p.o.

Followed by a combination treatment with:

1. PEG-interferon alfa-2b (PegIntron®): 1.5µg/kg bodyweight s.c./week (independent of genotype) plus
2. Ribavirin (Rebetol®):
   BW <65 Kg: 800 mg QD (2 capsules in the morning + 2 capsules in the evening)
   BW 65-85 Kg: 1,000 mg QD (2 capsules in the morning + 3 capsules in the evening)
   BW >85 Kg: 1,200 mg QD (3 capsules in the morning + 3 capsules in the evening)
3. AdoMet 3 x 400mg/d p.o.
4. Betaine 2 x 3g/d p.o.

Duration of treatment: will depend on HCV-genotype

- 24 weeks for genotypes 2 and 3
- 48 weeks for genotypes 1, 4 and 5

For patients, who do not show an adequate response at week 12 (adequate response at week 12 = drop of HCV RNA of at least 2log or HCV RNA-negativity), treatment can be stopped and the patient enters follow up phase.

Duration of study: Will include the treatment period (24 or 48 weeks) as well as the follow-up period of 24 weeks (totally: 48 or 72 weeks)

10.3. Treatment Group B Patients:

Patients will receive one week of treatment with PegIntron and Rebetol, followed by a combination treatment with PegIntron, Rebetol, AdoMet and betaine. The dosages are identical to group A.

10.4. Dose Modification (for both group A and group B):

Dose modifications should be done as outlined in section 12.2.1.

11. Study Visits and Evaluations:

Appendix 1 defines the planned study visits and evaluations.

Unscheduled visits may be performed if required for assessment of laboratory parameters or clinical safety. An unscheduled visit must be performed within one week of the last visit for patients who have a hemoglobin decrease more than 2 g/dl since the entry visit or decreased to below 10 g/dl. At this new visit, a hematology sample should be obtained for analysis and, if the hemoglobin value still meets the above criteria, the dose reduction/discontinuation and additional monitoring visits will apply.

12. Adverse Events and Safety

An adverse event is any untoward medical occurrence or unfavorable and unintended sign in a subject administered a pharmaceutical product, biologic (at any dose), or medical device, **whether or not considered related to the use of that product**. This includes the onset of new illness and the exacerbation of pre-existing conditions.

Additionally, any event that is associated with or observed in conjunction with a product overdose (whether accidental or intentional) or a product abuse and/or withdrawal is also considered an adverse event.

All adverse events must be recorded in the subject's medical records and on the case report form. The onset and end dates, severity and relationship to study drug will be recorded for each adverse event. The severity of the adverse event will be assessed according to specific guidelines (Section 12.1). Any action or outcome (e.g., hospitalization, discontinuation of therapy, etc.) will also be recorded for each adverse event.

Subjects will be questioned and/or examined by the investigator or his/her designee for evidence of adverse events. The questioning of subjects with regard to the possible occurrence of adverse events will be generalized such as, "How have you been feeling since your last visit?" The presence or absence of specific adverse events should not be elicited from subjects.

12.1 Assessment of Adverse Event Severity and Relationship to Treatment

The modified World Health Organization (WHO) grading system will be used for grading severity of AEs (appendix 2). For AEs not covered by the modified WHO grading system, the following definitions will be used:

The following definitions will be used for grading the severity of adverse events:

Mild:	awareness of sign, symptom, or event, but easily tolerated
Moderate:	discomfort enough to cause interference with usual activity and may warrant intervention
Severe:	incapacitating with inability to do usual activities or significantly affects clinical status, and warrants intervention
Life-threatening:	immediate risk of death

The investigator must also assess the relationship of any adverse event to the use of study drug, based on available information, using the following guidelines:

Unlikely related:	no temporal association, or the cause of the event has been identified; or the drug, biological, or device cannot be implicated
Possibly related:	temporal association, but other etiologies are likely to be the cause; however, involvement of the drug, biological, or device cannot be excluded
Probably related:	temporal association; other etiologies are possible, but unlikely

12.2 Monitoring Adverse Events

Subjects having adverse events will be monitored with relevant clinical assessments and laboratory tests, as determined by the investigator. All adverse events must be followed to satisfactory resolution or stabilization of the event(s).

Any actions taken and follow-up results must be recorded either on the appropriate page of the case report form, as well as in the subject's source documentation. Follow-up laboratory results should be filed with the subject's source documentation.

For all adverse events that require the subject to be discontinued from the study, relevant clinical assessments and laboratory tests must be repeated at clinically appropriate intervals until final resolution or stabilization of the event(s).

12.2.1 Management of Adverse Events

Subjects having AEs will be monitored with relevant clinical assessments and laboratory tests, as determined by the investigator. The investigator is expected to report ongoing AEs at completion of the clinical study to the primary care physician who will determine the need for and provide standard medical care.

Any actions taken and follow-up results must be recorded either on the appropriate page of the CRF or in a follow-up letter to ESSEX Chemie AG as well as in the subject's source documentation. Follow-up laboratory results should be filed with the subject's source documentation.

For all AEs that require the subject to be discontinued from the study, relevant clinical assessments and laboratory tests will be repeated on at least a monthly basis until final resolution or stabilization of the event(s).

Adverse events can be managed by dose reduction of one or both study medications. In the case of life-threatening AEs as described in Section 12.2.4 both PEG-Intron plus REBETOL must be discontinued permanently.

Reduced doses will be achieved by decreasing the injection volume of the PEG-Intron and the number of capsules/tablets of REBETOL as referenced in Table 1 and Table 2. While at a reduced dose level, the subject should return for assessment at a minimum of every two weeks until the AE resolves or the subject is stable. If the AE persists but does not fall into the range for permanent discontinuation, the reduced dose of PEG-Intron and/or ribavirin (whichever has been reduced) may be continued. At the discretion of the investigator, the dose of PEG-Intron and/or ribavirin may be increased to full dose, directly or in steps, when the AE subsides. If the AE recurs, the subject should be maintained at the previously tolerated reduced dose or, at the discretion of the investigator, discontinue treatment.

Table 1 Dose Reduction for REBETOL

Screening Weight[a]	Full Daily Dose	First Reduced Dose	First Reduced Number of Capsules	Second Reduced Dose	Second Reduced Number of Capsules
40-65 kg	800 mg/day	600 mg/day	1 in AM/2 in PM	400 mg/day	1 in AM/1 in PM
>65-85 kg	1000 mg/day	800 mg/day	2 in AM/2 in PM	600 mg/day	1 in AM/2 in PM
>85-105 kg	1200 mg/day	1000 mg/day	2 in AM/3 in PM	800 mg/day	2 in AM/2 in PM
>106-125 kg	1400 mg/day	1000 mg/day	2 in AM/3 in PM	800 mg/day	2 in AM/2 in PM

a: Use standard rounding procedures: for 0.1-0.4 kg, round down and 0.5-0.9 kg, round up.

Table 2: PEG-Intron : Weekly Reduced Dosing : Redipen

	1.0 μg /kg		0.5 μg /kg	
Body Weight	Vial/Pen Strength	Administer Once weekly (ml)	Vial/Pen Strength	Administer once Weekly (ml)
30-35 kg	50	0.3	50*	0.15
36-45 kg	50	0.4	50*	0.2
46-56 kg	50	0.5	50*	0.25
57-72 kg	80	0.4	50	0.3
73-88 kg	80	0.5	50	0.4
89-106 kg	100	0.5	50	0.5
>106kg**	120	0.5	80	0.4

* Must use vial. Minimum delivery for pen is 0.3 ml.
** For patients > 120 kg, use 80 μg/0.5 ml vial

12.2.2 Management of Selected Hematologic and Biochemical Parameters

Based on the side effect profile of pegylated interferon alfa and ribavirin there are selected hematologic and biochemical parameters (listed in Table 3) that have specific requirements for dose reduction or permanent discontinuation of therapy. All other hematologic and biochemical parameters will be managed using the Grades 1-4 guidelines in Appendix 2.

Table 3 Dose Reduction for Selected Hematologic and Biochemical Parameters

	Dose Reduction (see Table 1-2)	Permanent Discontinuation of Treatment PEG-Intron and REBETOL
Hemoglobin	<10 g/dL (Rebetol)[a]	<8.5 g/dL
White Blood Count	<1.5 x 10^9/L (PEG-Intron)[a]	<1.0 x 10^9/L
Neutrophil Count	<0.75 x 10^9/L (PEG-Intron)[a]	<0.5 x 10^9/L
Platelet Count	<50 x 10^9/L (PEG-Intron)[a]	<25 x 10^9/L
Bilirubin – Direct		2.5 x upper limit of normal
Bilirubin – Indirect	>5 mg/dL (>85.5 μmol/L) (Rebetol)[a]	>4 mg/dL (>68.4 μmol/L) (for >4 weeks)
Creatinine		>2.0 mg/dL (>176.8 μmol/L)
ALT/AST		2 x baseline and >10 x upper limit of normal

a: Study medication to be reduced.

If a subject meets the criteria for dose reduction, apply the first dose reduction level. An additional visit and blood sample analysis will be scheduled. If the first dose reduction does not improve the hematologic or biochemical results, a second dose reduction will apply.

12.2.3 Management of Depression During Study

Subjects who develop mild depression may continue their study medication and should be monitored weekly (by visit or by phone) for four to eight weeks. If the subject's status is stable, the subject may resume the normal visit schedule with instructions to call the investigator immediately if the subject feels that the depression has worsened. If the subject's condition worsens, see instructions for moderate and/or severe depression below.

Subjects with moderate depression should have their dose of PEG-Intron reduced according to Tables 1-2. Subjects are to be monitored weekly (by visit or phone, at least two visits should be in the office) for four to eight weeks (depending on the subject's status) to assure that their status is stable. These subjects may remain on reduced PEG-Intron dosing if the condition is considered stable and does not interfere with the subject's normal activities. Other clinical management intervention may be instituted as necessary. Subjects will be instructed to call the investigator immediately if they feel their depression has worsened. If the subject's symptoms do not improve but are stable for four weeks, the investigator should consider psychiatric consultation. If the subject's condition worsens, the subject should immediately discontinue both drugs and the investigator should make a priority assessment of the severity of the subject's condition. Appropriate psychotherapeutic measures should be instituted and the subject should be followed weekly or biweekly (depending on the investigator's clinical judgment) by visit or by phone until the subject's status has returned to baseline conditions.

A subject with severe depression according to the criteria outlined by the Diagnostic and Statistical Manual of Mental Disorders (DSM-IV™ , Appendix 3) or with suicidal ideation/attempt will discontinue all study medications permanently and will obtain psychiatric consultation immediately by referral to a psychiatrist by the investigator, until depressive symptoms have moderated and improved to baseline conditions. The subject should not resume therapy and should be followed until resolution of the symptoms.

12.2.4 Life Threatening Adverse Events

A subject who develops a life threatening event must have their PEG-Intron plus REBETOL therapy discontinued permanently. The subject should return for a follow-up evaluation(s) as clinically indicated or in a maximum of two weeks and should remain under medical observation until the AE resolves or the subject is stable.

12.3 Known Adverse Events Relating to the Underlying Clinical Condition

General medical complaints typically attributed to CHC consist of fatigue, right upper quadrant pain, flu-like symptoms, and headache. Hepatic manifestations of CHC include cirrhosis and evidence of liver decompensation, such as bleeding esophageal varices, coagulopathy, thrombocytopenia, jaundice, hypoproteinemic states with resultant ascites, edema, or anasarca and hepatic encephalopathy. Hepatocellular carcinoma is also known to occur as a complication of longstanding CHC. Extra-hepatic manifestations of CHC include cryoglobulinemia, membranous and membranoproliferative glomerulonephritis, peripheral neuropathy, Raynaud's Syndrome, vasculitis, and porphyria cutanea tarda. Less well confirmed potential associations with CHC are polyarteritis nodosa, Sjogren's Syndrome, and Lichen Planus.

12.4 Known Potential Toxicities of Study Drug

AEs known to be associated with the study drugs still need to be recorded in the subject's medical records and on the CRF regardless of causality. Refer to the Investigator's Brochure and/or product labels for a summary of AEs observed to date.

12.5 Definition of Serious Adverse Events

A serious adverse event (SAE) is any adverse drug or biologic or device experience occurring at any dose that results in any of the following outcomes:

- death;
- life-threatening AE (ie, one that places the subject, in the view of the initial reporter, at immediate risk of death from the AE as it occurs);
- persistent or significant disability/incapacity;
- required in-patient hospitalization, or prolonged hospitalization;
- cogenital anomaly or birth defect.

Additionally, important medical events that may not result in death, be life-threatening, or require hospitalization may be considered a serious adverse event when, based upon appropriate medical judgment, they may jeopardize the subject and may require medical or surgical intervention to prevent one of the outcomes listed in this definition. Examples of such medical events include allergic bronchospasm requiring intensive treatment in an emergency room or at home, blood dyscrasias or convulsions that do not result in in-patient hospitalization, or the development of drug dependency or drug abuse.

In addition, laboratory-value(s) changes may require reporting unless otherwise specified in this section of the protocol.

Grade 4 laboratory abnormalities that are not accompanied by clinical manifestations will NOT be considered SAEs. Out of range liver functional tests are not unexpected in subjects with CHC and do not necessarily have to be considered important medical events, unless there is a significant elevation from baseline or evidence of hepatic failure.

All SAEs, whether or not deemed drug-related or expected, must be reported by the principal investigator or designee to Essex Chemie AG (attn. Safety Officer; phone: 041 368 49 19, fax: 041 368 49 79), within 24 hours (one working day) of first becoming aware of the event. If the report is given to the Safety Officer via telephone rather than in writing on the form designated for SAE reporting, the telephone report must provide a full description of the event and any sequelae, including the investigator-determined causality to study drug so that the appropriate written report can be completed by the Safety Officer.
SAEs will be reported by Essex Chemie AG to Swissmedic and by the study investigators to the appropriate ethical commission within 7 days.
SAEs that occur at any time after the inclusion of the subject in the study (defined as the time when the subject signs the informed consent) until the week 24 follow-up visit must be reported. The subject is considered to have completed the study

EITHER after the completion of the last visit or contact (eg, phone contact with the investigator or designee) OR after the last dose of the study medication, whichever is later. The date of discontinuation is when a subject and/or investigator determines that the subject can no longer comply with the requirements for any further study visits or evaluations. In the specific circumstance of screen failures, SAEs should be collected from the time of consent signing until the subject is considered a screen failure.

12.6 Reporting of Subject Death

The death of any subject during the study until the 24-week follow-up visit, regardless of the cause, must be reported to Essex Chemie AG (attn. Safety Officer; phone: 041 368 49 19, fax: 041 368 49 79), within 24 hours of the primary investigator or designee first becoming aware of the death. If the report is made via telephone rather than in writing on the form designated for serious adverse event reporting, then the telephone report must include a full description of the circumstances, including the investigator-determined causality to study drug so that the appropriate written report can be completed by the Safety Officer.

If an autopsy is performed, the report must be provided to ESSEX Chemie AG.

Reports of all deaths related to the study drugs must be reported as soon as possible and within 7days by Essex Chemie AG to Swissmedic and by the study investigators to the appropriate ethical commission.

12.7 Early Termination

If a patient is removed from the study prior to completion of the therapy, the reason for doing so and the date the patient is discontinued will be documented in the patient's medical record. The date of last dose of Peg-Intron and Rebetol must also be documented.
If a patient is removed from the study, the clinical and laboratory evaluations, which would have been performed at the last treatment visit, should be obtained.
If the patient is removed because of intolerance to one of the study medications, the patient should remain under medical observation until resolution or stabilization of the adverse event. A patient will be removed from the study in case of serious or life-threatening adverse event, pregnancy, explicit wish to withdraw, failure to comply with the study requirements, or in case the investigator feels that it is in the best interest of the patient.
The occurrence of an adverse event causing the discontinuation of treatment will not be considered as a reason for removal from the study but will advance the patient to the follow-up phase, during which the event will be followed until resolution.

12.8 Reporting of Pregnancies

Although not considered a serious adverse event (unless an event occurs with a serious outcome), pregnancy information on clinical study subjects is considered a Reportable Event and is collected by Essex Chemie AG's Drug Safety Surveillance (DSS) department. If a subject or the female partner(s) of a male study subject should become pregnant during the course of the study, the principal investigator or designee must contact Essex Chemie AG (attn. Safety Officer; phone: 041 368 49 19, fax: 041 368 49 79), within five working days of the principal investigator or designee first becoming aware of the pregnancy. If a serious adverse event occurs in conjunction with the pregnancy, then the reporting time frame for a serious adverse event (one working day) must be met. Follow-up information on the outcome of the pregnancy should also be forwarded to Essex Chemie AG.

12.9 Preplanned Hospitalizations or Procedures

During the study, if a subject has a hospitalization or procedure (e.g., elective surgery) that was scheduled prior to the subject entering the study (i.e., before the subject signed the informed consent) for an event/condition that occurred before the study, the hospitalization is considered a therapeutic intervention and not the result of a serious adverse event. However, if the event/condition worsens during the course of the study it must be reported as an adverse event (or serious adverse event, if the event/condition results in a serious outcome such as hospitalization).

12.10 Reports of Overdose

An overdose of PEG-Intron is defined as a dose that exceeds twice the expected injection volume per protocol. An overdose of ribavirin is defined as that which exceeds 20 mg/kg/day. Any event associated with or observed in conjunction with a product overdose (whether accidental or intentional) or a product abuse and/or withdrawal is considered an AE. Although overdose is no longer considered an SAE by FDA (unless an event occurs with a serious outcome), information on overdoses in clinical subjects is collected by Essex Chemie AG's DSS department. Should a subject experience an overdose (as defined above) during the course of the study, the principal investigator or designee must contact Essex Chemie AG (attn. Safety Officer; phone: 041 368 49 19, fax: 041 368 49 79), within five working days of the principal investigator or designee first becoming aware of the overdose (if a serious AE occurs in conjunction with the overdose, then the reporting time frame for an SAE must be met). Follow-up information should also be forwarded to Essex Chemie AG.

12.11 Protocol-Specific Exceptions to Serious Adverse Event Reporting

This study does not contain protocol-specific exceptions to adverse event reporting.

12.12 Reporting of Investigational Medicinal Product Quality Complaints

Any defect or possible defect in an investigational medicinal product (defined as a pharmaceutical form of an active substance or placebo being tested or used as a reference in a clinical trial) must be reported by the principal investigator or designee to Essex Chemie AG within one working day of first becoming aware of the possible defect. This report to Essex Chemie AG may be made by telephone or by faxing the Investigational Medicinal Product Quality Complaint (IMPQC) Form to Essex Chemie AG (attn. Safety Officer; phone: 041 368 49 19, fax: 041 368 49 79). The product and packaging components in question, if available, must be stored in a secure area under specified storage conditions until it is determined whether the product is required to be returned for investigation of the defect. If the product complaint is associated with an SAE, the SAE must be reported separately in accordance with the protocol, and the SAE report should mention the product quality complaint.

References:

1. Fried MW, Shiffman ML, Reddy KR, Smith C, Marinos G, Goncales FL, Jr., Haussinger D, et al. Peginterferon alfa-2a plus ribavirin for chronic hepatitis C virus infection. N Engl J Med 2002;347:975-982.
2. Manns MP, McHutchison JG, Gordon SC, Rustgi VK, Shiffman M, Reindollar R, Goodman ZD, et al. Peginterferon alfa-2b plus ribavirin compared with interferon alfa-2b plus ribavirin for initial treatment of chronic hepatitis C: a randomised trial. Lancet 2001;358:958-965.
3. Yoneyama M, Kikuchi M, Natsukawa T, Shinobu N, Imaizumi T, Miyagishi M, Taira K, et al. The RNA helicase RIG-I has an essential function in double-stranded RNA-induced innate antiviral responses. Nat Immunol 2004;5:730-737.
4. Yoneyama M, Suhara W, Fukuhara Y, Fukuda M, Nishida E, Fujita T. Direct triggering of the type I interferon system by virus infection: activation of a transcription factor complex containing IRF-3 and CBP/p300. Embo J 1998;17:1087-1095.
5. Lin R, Heylbroeck C, Pitha PM, Hiscott J. Virus-dependent phosphorylation of the IRF-3 transcription factor regulates nuclear translocation, transactivation potential, and proteasome-mediated degradation. Mol Cell Biol 1998;18:2986-2996.
6. Heim MH. RIG-I: an essential regulator of virus-induced interferon production. J Hepatol 2005;42:431-433.
7. Sato M, Suemori H, Hata N, Asagiri M, Ogasawara K, Nakao K, Nakaya T, et al. Distinct and essential roles of transcription factors IRF-3 and IRF-7 in response to viruses for IFN-alpha/beta gene induction. Immunity 2000;13:539-548.
8. Sharma S, tenOever BR, Grandvaux N, Zhou GP, Lin R, Hiscott J. Triggering the interferon antiviral response through an IKK-related pathway. Science 2003;300:1148-1151.
9. Fitzgerald KA, McWhirter SM, Faia KL, Rowe DC, Latz E, Golenbock DT, Coyle AJ, et al. IKKepsilon and TBK1 are essential components of the IRF3 signaling pathway. Nat Immunol 2003;4:491-496.
10. Darnell JE, Jr, Kerr IM, Stark GR. Jak-STAT pathways and transcriptional activation in response to IFNs and other extracellular signaling proteins. Science 1994;264:1415-1421.
11. Heim MH. Intracellular signalling and antiviral effects of interferons. Dig Liver Dis 2000;32:257-263.
12. Foy E, Li K, Sumpter R, Jr., Loo YM, Johnson CL, Wang C, Fish PM, et al. Control of antiviral defenses through hepatitis C virus disruption of retinoic acid-inducible gene-I signaling. Proc Natl Acad Sci U S A 2005;102:2986-2991.
13. Li K, Foy E, Ferreon JC, Nakamura M, Ferreon AC, Ikeda M, Ray SC, et al. Immune evasion by hepatitis C virus NS3/4A protease-mediated cleavage of the Toll-like receptor 3 adaptor protein TRIF. Proc Natl Acad Sci U S A 2005;102:2992-2997.
14. Heim MH, Moradpour D, Blum HE. Expression of hepatitis C virus proteins inhibits signal transduction through the jak-STAT pathway. J Virol 1999;73:8469-8475.
15. Meraz MA, White JM, Sheehan KCF, Bach EA, Rodig SJ, Dighe AS, Kaplan DH, et al. Targeted disruption of the Stat1 gene in mice reveals unexpected physiologic specificity in the Jak-Stat signaling pathway. Cell 1996;84:431-442.

16. Durbin JE, Hackenmiller R, Simon MC, Levy DE. Targeted disruption of the mouse Stat1 results in compromised innate immunity to viral disease. Cell 1996;84:443-450.
17. Dupuis S, Jouanguy E, Al-Hajjar S, Fieschi C, Al-Mohsen IZ, Al-Jumaah S, Yang K, et al. Impaired response to interferon-alpha/beta and lethal viral disease in human STAT1 deficiency. Nat Genet 2003;33:388-391.
18. Duong FH, Filipowicz M, Tripodi M, La Monica N, Heim MH. Hepatitis C virus inhibits interferon signaling through up-regulation of protein phosphatase 2A. Gastroenterology 2004;126:263-277.
19. Duong F, Christen V, Heim MH. S-Adenosylmethionine and Betaine Improve Interferon Signaling in Human Hepatoma Cells Expressing Hepatitis C Virus Proteins or Containing a Hepatitis C Virus Replicon. submitted 2005.
20. Mowen KA, Tang J, Zhu W, Schurter BT, Shuai K, Herschman HR, David M. Arginine methylation of stat1 modulates ifnalpha/beta-induced transcription. Cell 2001;104:731-741.
21. Liu B, Mink S, Wong KA, Stein N, Getman C, Dempsey PW, Wu H, et al. PIAS1 selectively inhibits interferon-inducible genes and is important in innate immunity. Nat Immunol 2004;5:891-898.
22. Shiffman ML, Di Bisceglie AM, Lindsay KL, Morishima C, Wright EC, Everson GT, Lok AS, et al. Peginterferon alfa-2a and ribavirin in patients with chronic hepatitis C who have failed prior treatment. Gastroenterology 2004;126:1015-1023; discussion 1947.

Appendix 1a: Schedule of Assessments in group A

	Baseline	At the end of 1 week of AdoMet-Betaine (pre-) treatment	During full combination treatment after 1, 2, 4 days, 1, 2, 4, 6, 8, 12 weeks, thereafter every 6 weeks until end of treatment	Follow-up 6 months after end of treatment
Medical history	X			
Adverse Events / Tolerability	X	X	X	X
Physical Exam / Vital Signs	X	X	X	X
Concomitant Medication	X	X	X	X
Hematology	X	X	X	X
Blood chemistry	X	X	X	X
Quick / INR	X			
AFP	X			
ANA	X			
TSH	X		X (a)	X
HBs Ag, HAV-IgM, HIV-test	X			
Liver biopsy	X (b)			
Urinanalysis	X			
Pregnancy Test	X		X (c)	X
HCV genotype	X			
HCV RNA quantitative	X	X	X (d)	X
Isolation of leucocytes and asservation of serum	X	X	X (e)	

(a) only at week 12, 24 and 48
(b) if not done within 12 months prior to study entry
(c) every 4 weeks
(d) at day 1, 2, 4, week 1, 2, 4, 8, 12, 24, 48
(e) at day 1, 2, 4 (24hours, 48hours, 96hours), week 1 after first injection of PegIntron, then weeks 2, 4, 6, 8, 12, 24, 48

Appendix 1b: Schedule of Assessments in group B

	Baseline	During treatment with PegIntron and Ribavirin (first week) after 1,2,4 days	During treatment after 1, 2, 4, 6, 8, 12 weeks, thereafter every 6 weeks until end of treatment	Follow-up 6 months after end of treatment
Medical history	X			
Adverse Events / Tolerability	X		X	X
Physical Exam / Vital Signs	X		X	X
Concomitant Medication	X		X	X
Hematology	X		X	X
Blood chemistry	X		X	X
Quick / INR	X			
AFP	X			
ANA	X			
TSH	X		X (a)	X
HBs Ag, HAV-IgM, HIV-test	X			
Liver biopsy	X (b)			
Urinanalysis	X			
Pregnancy Test	X		X (c)	X
HCV genotype	X			
HCV RNA quantitative	X	X (e)	X (d)	X
Isolation of leucocytes and asservation of serum	X	X	X (f)	

(a) only at week 12, 24 and 48
(b) if not done within 12 months prior to study entry
(c) every 4 weeks
(d) at the end of weeks 1, 2, 4, 8, 12, 24, 48
(e) at the end of the first week (PegIntron-Rebetol only)
(f) at day 1, 2, 4 (24hours, 48hours, 96hours), week 1 after first injection of PegIntron, then weeks 2, 4, 6, 8, 12, 24, 48

Appendix 3: Criteria For Major Depressive Episode / DSM-IV™

A. Five (or more) of the following symptoms have been present during the same two-week period and represent a change from previous functioning; at least one of the symptoms is either (1) depressed mood or (2) loss of interest or pleasure.

Note: Do not include symptoms that are clearly due to a general medical condition, or mood-incongruent delusions or hallucinations.

1. Depressed mood most of the day, nearly every day, as indicated by either subjective report (e.g., feels sad or empty) or observation made by others (e.g., appears tearful). **Note:** In children and adolescents, can be irritable mood.

2. Markedly diminished interest or pleasure in all, or almost all, activities most of the day, nearly every day (as indicated by either subjective account or observation made by others)

3. Significant weight loss when not dieting or weight gain (e.g., a change of more than 5% of body weight in a month), or decrease or increase in appetite nearly every day. **Note:** In children, consider failure to make expected weight gains.

4. Insomnia or hypersomnia nearly every day

5. Psychomotor agitation or retardation nearly every day (observable by others, not merely subjective feelings of restlessness or being slowed down)

6. Fatigue or loss of energy nearly every day

7. Feelings or worthlessness or excessive or inappropriate guilt (which may be delusional) nearly every day (not merely self-reproach or guilt about being sick)

8. Diminished ability to think or concentrate, or indecisiveness, nearly every day (either by subjective account or as observed by others)

9. Recurrent thoughts of death (not just fear of dying), recurrent suicidal ideation without a specific plan, or a suicide attempt or a specific plan for committing suicide

B. The symptoms do not meet criteria for a Mixed Episode (see p. 335).[1]

C. The symptoms cause clinically significant distress or impairment in social, occupations, or other important areas of functioning.

---

[1] Not applicable to this protocol.

D. The symptoms are not due to the direct physiological effects of a substance (e.g., a drug of abuse, a medication) or a general medical condition (e.g., hypothyroidism).

E. The symptoms are not better accounted for by bereavement (i.e., after the loss of a loved one), the symptoms persist for longer than 2 months or are characterized by marked functional impairment, morbid preoccupation with worthlessness, suicidal ideation, psychotic symptoms, or psychomotor retardation.

Appendix 4: Randomization List

Genotype 1,4

Group	Date	Patient-Initials
A		
A		
B		
A		
B		
B		
B		
A		
A		
B		
B		
B		
A		
B		
A		
A		
A		
A		
B		
B		

Genotype 2,3

Group	Date	Patient-Initials
B		
A		
A		
B		
A		
B		
B		
A		
B		
A		
A		
B		
B		
A		
B		
B		
A		
B		
A		
A		
